# Oviposition, Life Cycle, and Longevity of the Leaf-Cutting Ant *Acromyrmex rugosus rugosus*

**DOI:** 10.3390/insects8030080

**Published:** 2017-08-04

**Authors:** Sandra S. Verza, Rosilda M. Mussury, Roberto S. Camargo, Ana Paula P. Andrade, Luiz C. Forti

**Affiliations:** 1 Laboratório de Insetos Sociais-Praga, Departamento de Produção Vegetal-Defesa Fitossanitária, Faculdade de Ciências Agronômicas, UNESP-São Paulo State University, Fazenda Experimental Lageado, Rua José Barbosa de Barros, 18610-307 Botucatu, São Paulo, Brazil; camargobt@hotmail.com (R.S.C.); anapaulaprotti@gmail.com (A.P.P.A.); luizforti@fca.unesp.br (L.C.F.); 2 Faculdade de Ciências Biológicas e Ambientais, Universidade Federal da Grande Dourados, UFGD, 79804-970 Dourados, Mato Grosso do Sul, Brazil; maramussury@ufgd.edu.br

**Keywords:** cannibalism, leaf-cutting ants, life cycle, oviposition, worker longevity

## Abstract

Studies related to the demography of individual members from ant colonies have received little attention, although they are the basis to understanding the population dynamics of colonies. Thus, the objective of this work was to study the queen oviposition rate and the duration of the life cycle and longevity of *Acromyrmex rugosus rugosus* workers. To determine the oviposition rate, queens from three colonies were individually placed in plastic containers, and the eggs laid were quantified over a 96 h period. The development of the immature forms was observed every 24 h, with which the duration of each stage of development was determined. To verify the longevity of workers, the newly emerged adults were marked and daily observations were made. According to the results, there is variation in the development time of immature forms within the colony itself and between colonies. In addition, the number of eggs deposited was also inconstant in the three colonies, ranging from 5 to 119 eggs per day, while the longevity of workers varied from 3 to 7 months. Occasionally, it was found that the workers feed on the eggs produced by the queen; besides, there was a disappearance of larvae and pupae during the research, indicating a possibility of the practice of cannibalism in this species.

## 1. Introduction

There are relatively few data on the life span and degree of survival of ants [1]. Studies related to oviposition rates, and the development of larvae, pupae, and adult leaf-cutting ants are also scarce in the literature [2]. However, studies related to egg laying, life cycle, and longevity are pertinent, and support basic studies in behavior and population ecology as well as studies applied to pest control.

Oviposition is a determinant factor to the growth of ant colonies, as long as increased oviposition rate raises the number of individuals in the colony [2,3]. In general, among social insects, the queen is in charge of reproduction, while workers engage in activities related to nest maintenance, defense, supply, and care of the offspring [4]. In leaf-cutting ants, at the beginning of colony formation, the queen is able to produce two types of eggs: feeding or trophic eggs and reproductive eggs [5,6,7].

After the foundation of the colony, it is assumed that the queen is solely responsible for the oviposition of fertile eggs, which results in the production of diploid workers, but which are sterile and incapable of mating, although the queen is also involved in the production of haploid males coming from unfertilized eggs [5,7]. However, workers of some species of *Atta* and *Acromyrmex*, including *Acromyrmex rugosus rugosus*, are not completely sterile, since they are able to produce reproductive eggs, which originate males, in the absence of the queen [8,9,10,11,12]. It is also known that feeding eggs are not produced by queens only, but also by workers [13,14].

The immature forms (eggs, larvae, and pupae) of all sizes and stages of development of most social Hymenoptera are immobile, and completely dependent on adult workers that provide for their needs. However, unlike wasps and social bees, the offspring of ants are kept in a collective nest rather than in individual cells, which prevents them from being properly cared for by workers. Besides the feeding, displacement, and cleaning of the offspring carried out by workers, larvae and pupae also depend on the help of workers in order to hatch from their eggs and emerge from the ecdyses during the moulting process [15,16,17,18,19,20,21]. Additionally, in *Acromyrmex*, workers are to some extent able to assess and respond to the individual needs of the larvae, which vary according to fasting time [21].

Therefore, immature stages depend on the social environment in order to develop, and in this sense, it is important to emphasize that caring for the offspring is the first task that workers take on in the course of their lives [1,20]. Almost all adult social insects undergo behavioral changes as they age, a fact that changes their role in society [1]. Thus, younger workers tend to perform tasks inside the nest, while older workers are committed to tasks outside the nest, such as foraging [4,22]. However, workers broaden their behavior repertoire without excluding behaviors they used to show early in their lives [23]. This age polyethism is related to an increase in the risk of mortality during activities outside the nests, and to an increase in the life expectancy of young workers when they are engaged in activities inside the nest [22].

It is worth remembering herein that leaf-cutting ants are considered, alongside certain termites, as the most important natural herbivores in many ecosystems, although they do not feed directly on the plants they cut [24], because they grow an obligate mutualist fungus with the material collected. This fungus produces a protein-rich structure called gongylidia, which is the primary food source for the developing offspring [8]. The fungus is also an important source of food for the queen and adult workers, meeting approximately 50% of the workers nutritional needs [25]. Thus, the fungus allows these ant species to develop colonies of greater complexity, both in the structure of nests and in the population and composition of castes, since this type of substrate increases the energy (activity) of the colony [26].

Bearing in mind that members of a society become more specialized within their roles as their society becomes more efficient, larger, and geometrically more structured [27], the objectives of this work were to determine the oviposition rate of the queen and the life cycle duration and longevity of *A. rugosus rugosus* workers.

## 2. Material and Methods

### 2.1. Colony Collection

The colonies of the *Acromyrmex rugosus rugosus* subspecies were collected in Botucatu, São Paulo, Brazil (22°53′09″ S; 48°26′42″ W). Excavations were done to collect the colonies, which were carefully placed in cylindrical acrylic containers with a one-centimeter layer of plaster at the bottom to maintain the fungus culture’s moisture, and taken to the Social Insects-Pest Laboratory, Faculty of Agronomic Sciences of São Paulo State University, Botucatu, São Paulo, Brazil.

### 2.2. Storage of the Colonies in the Laboratory

In the laboratory, the acrylic containers were interconnected by plastic tubes, where on one side the container (chamber) was connected for the placement of dicotyledonous leaves (substrate), and on the other side for the depleted fungal material (waste). These colonies were kept under a controlled temperature of 24 ± 2 °C and a relative humidity of 70% ± 20%.

### 2.3. Queen Oviposition

Before starting the bioassay, the fungus volume of the original colony was measured, and then, with the aid of tweezers, queens from three colonies of *A. rugosus rugosus* were individualized in plastic containers (sub-colonies) with the edges smeared with Fluon (fluorene resin), a product that prevents the escape of the ants. Later, based on the methodology of Marinho and Della Lucia (1998) [2], groups with five small workers and five medium-sized workers were selected from the substrate chambers, and placed in each container for the care and cleaning of their respective queens, together with a standardized amount of fungus culture (15 mL) to feed them.

The number of eggs laid during a period of 96 h was observed at 24 h intervals, because after this period the queens interrupted oviposition. Following the methodology of Andrade (2002) [28], every 24 h the queen and the workers were transferred to another container with a 15 mL portion of fungus garden to continue the experiment, totalizing four replicates or sub-colonies (24, 48, 72, and 96 h) for each colony. Then, with the aid of a stereomicroscope, the portions of fungus where the queens were staying were carefully examined to quantify the number of eggs laid.

### 2.4. Workers’ Life Cycle (Egg Period until the Workers’ Emergence)

In order to provide care during development and to assist in the emergence of the adults, five additional garden workers and five waste workers were selected and placed in the containers with portions of fungus and eggs newly oviposited by the queens of the previous experiment. These containers were then placed in a BOD (biochemical oxygen demand) type incubator, at controlled temperature (24 °C), in accordance with the methodology of Andrade (2002) [28]. Every 24 h, the development of the eggs was observed, which allowed for the determination of the duration of each stage of development: (i) egg, (ii) larva, (iii) pupa, and (iv) adult worker, using a stereomicroscope, and with the aid of the latter, pieces of symbiotic fungus were carefully observed, cleaned, and offered to the workers for their nutrition.

### 2.5. Workers’ Longevity

To determine the workers’ longevity, the newly emerged adults of the previous experiment were marked with a fine-tipped brush with a small dot of ink in the pronotum using non-toxic permanent ink pens (EDDING 751 brand), due to its excellent adhesion, quick drying, and good visibility. Following the methodology of Andrade (2002) [28], different colors were used for each day of emergence.

Marked workers were returned to the fungal chamber (original) of their respective nests, and daily observations were made in all of the chambers of the nests, and, weekly, the waste was removed and observed under a stereomicroscope to quantify the number of marked workers who were dead.

### 2.6. Data Analysis

The mean and the standard error of the mean (Univariate procedure, SAS^®^) [29] were calculated for the queen daily oviposition rate and worker life cycle duration variables. In order to verify correlations between the variables number of eggs and number of workers emerged, the Pearson correlation test was performed at a 5% probability level. With the longevity time results of adult workers, a survival analysis (Lifetest procedure, SAS^®^) [29] was performed. To determine the non-parametric estimates of the survival distribution function, the Kaplan-Meier estimators of the distribution were used, and with them the Savage test (Log Rank) was carried out at a 5% probability level for comparison between nests. To describe the survival function results, the average duration time was considered, that is, the time to death of 50% of the workers, because as it is a non-parametric analysis this location parameter is the most appropriate one.

## 3. Results

### 3.1. Queen Oviposition

**Figure 1:**
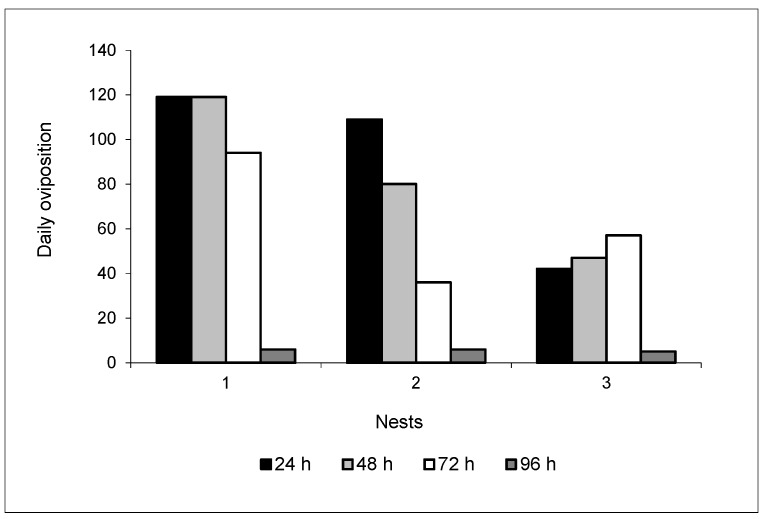
Daily oviposition of queens from three nests of *Acromyrmex rugosus rugosus*. Temperature: 24 °C.

The number of eggs deposited was very variable in the three colonies studied, ranging from 5 to 119 eggs per day. During the observations, it was seen in nests 1 and 2 that the number of eggs deposited reduced every 24 h. However, all of the nests presented a lower rate in the number of eggs in the last oviposition (Figure 1). After the 96 h period, the queens ceased their oviposition, and with this were returned to their nests of origin.

The nests 1, 2, and 3 used in the present experiment had different sizes of fungus culture, being 850, 450, and 225 mL, respectively. It was observed that the queen of the largest nest (fungus volume) was the one that deposited the highest average oviposition rate in 96 h (84.5), while the queen of the smallest nest oviposited the lowest number of eggs during the same period (37.8); the queen of nest 2, in turn, presented an average rate of 57.8 eggs (Table 1).

The average number of eggs/hour oviposited in the 96 h period by the queens of nests 1, 2, and 3 was on average 3.5, 2.4, and 1.6 eggs/h, respectively (Table 1), with the total mean being 2.5 eggs/h.

### 3.2. Workers’ Life Cycle

**Figure 2:**
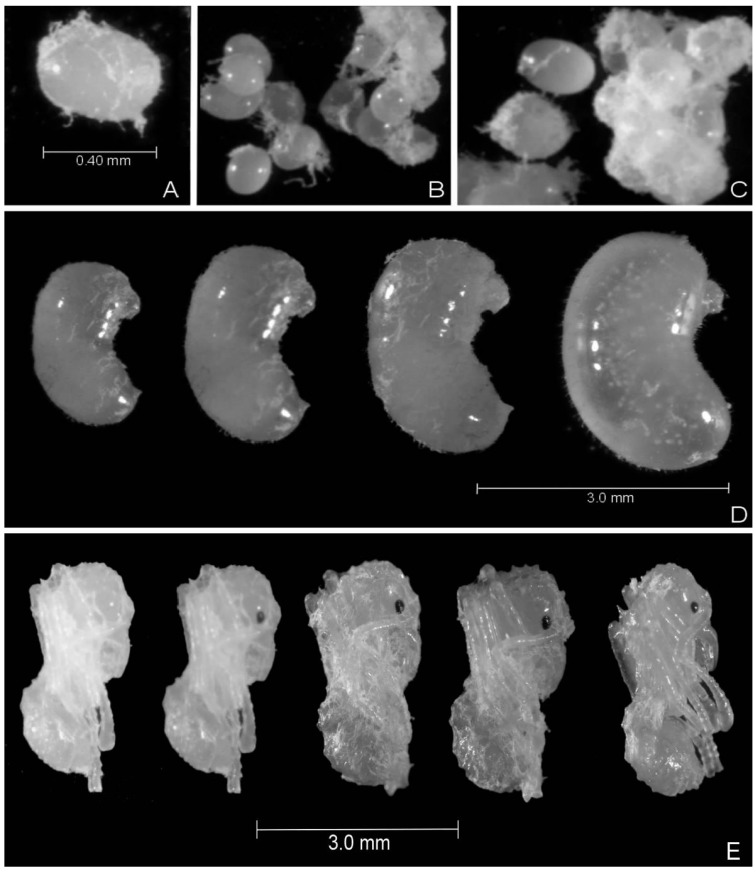
*Acromyrmex rugosus rugosus* offspring covered by mycelia of the symbiotic fungus. (**A**) Egg shape; (**B**) and (**C**) Grouped eggs; (**D**) Developing larvae, distention in the body and increase in the capacity of the integument indicate the end of the stage; (**E**) Pupae from the beginning of development (white) until near the adult period (dark).

The eggs found were grouped and covered by mycelia of the symbiotic fungus, just as were some larvae and most of the pupae (Figure 2). Some eggs had a thinner and longer shape, different from most. However, regardless of the format, some of the observed eggs were not viable, as they did not hatch. It was also observed that the workers sometimes fed on the eggs.

**Figure 3:**
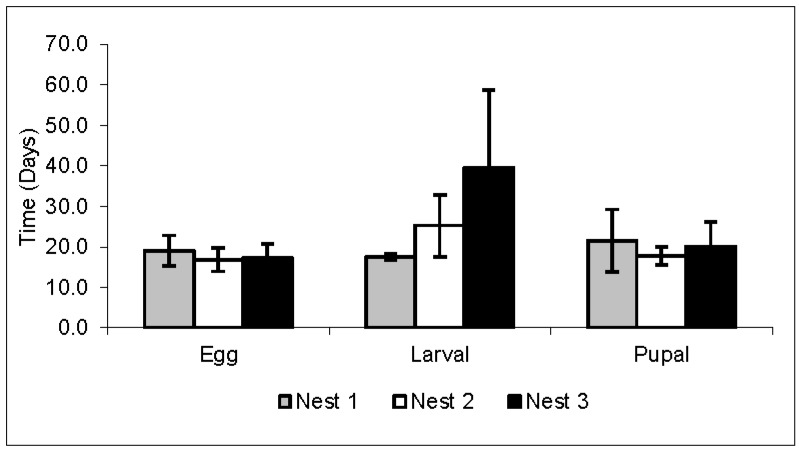
Stages. Mean and standard deviation of the duration time (days) of egg, larval, and pupal phases of *Acromyrmex rugosus rugosus* workers in three nests. Temperature: 24 °C.

**Figure 4:**
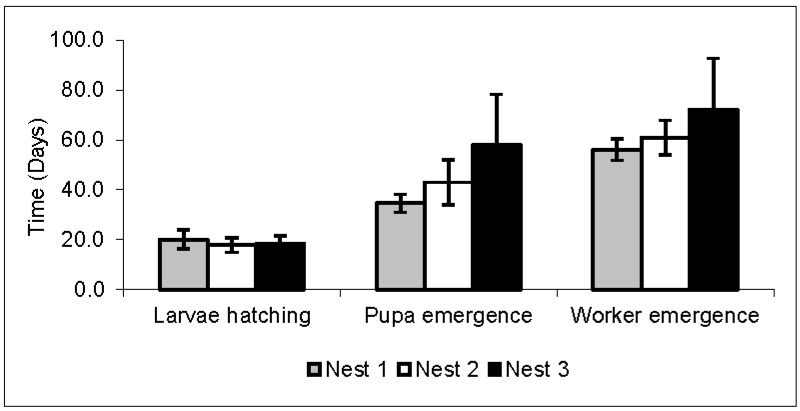
Average time and standard deviation for the hatching of the larva, and the emergence of the pupa and worker (adult) of *Acromyrmex rugosus rugosus* in three nests. Temperature: 24 °C.

The egg stage had an average duration of 17.7 days, the larval phase 27.4 days, and the pupal phase 19.8 days (Table 2 and Figure 3). The average development time (the time spent since egg deposition until the occurrence of hatching and emergence) for larval hatching was 18.7 days, and for the emergence of the pupa, 45.1 days. Thus, the average development time of *A. rugosus rugosus* workers was 62.9 days (Table 2 and Figure 4). These data were obtained from the mean of the four replicates (24, 48, 72, and 96 h) from the daily oviposition of each nest used in this study. It was not possible to detect significant changes in larvae that could indicate differences between larval instars. In addition, it was observed that many larvae and pupae did not develop.

There was variation in the development time in the larva and pupa phases (Figure 3), and consequently in the emergence of the pupa and adults (Figure 4) in the three nests, but the incubation period (egg stage duration) had little variation (Figure 3). The larval phase in nest 3 showed great variation inside it and when compared to the other two nests (Figure 3). The young forms were kept under a controlled temperature (24 °C) in order to avoid fluctuations in the development times of each phase, but variations still occurred.

The number of emerged workers in the three nests did not show significant correlation with the number of eggs laid by the queens (R^2^ = −0.6216, *p* = 0.5729^ns^).

### 3.3. Longevity

The workers’ life span was quite variable between colonies, ranging from 3 to 7 months (Table 3). The number of workers that successfully emerged in the previous experiment were followed to assess their longevity, and the sample size is represented in Table 3.

**Figure 5:**
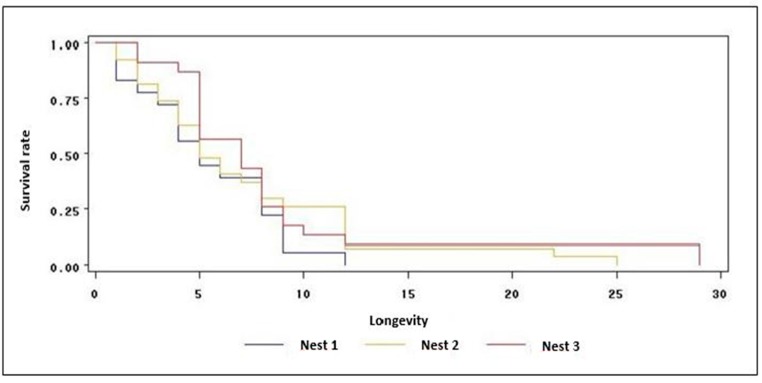
Survival analysis. Longevity survival function (weeks) of the *Acromyrmex rugosus rugosus* workers that emerged in three nests with different fungus volumes. Room temperature of 24 ± 2 °C and relative humidity of 70% ± 20%.

In relation to the workers’ mortality, a high rate was registered in the first weeks in the three nests, meaning a significant initial reduction in the population. Thus, the average time required for the death of 50% of the individuals was 5 weeks for nest 1 and 2, and 7 weeks for nest 3 (Table 3). The survival rate became stable in the last weeks of observation (Figure 5). The longevity of the workers did not present significant difference between colonies by the Savage Test (survival analysis) (χ^2^ = 2.2567; *p* = 0.3236^ns^). The highest longevity time was found for the workers in nest 3, which survived for a period of 29 weeks (Figure 5).

## 4. Discussion

In the present study, the oviposition rate of *Acromyrmex rugosus rugosus* queens was very variable in the three colonies. In addition, it was observed that the queen of the largest nest (fungus volume) had a higher average oviposition rate over 96 h, while the queen of the smallest nest had a lower number of eggs during this same period. These results were different from those showed by other *Acromyrmex* species already studied [2,28]. In *Acromyrmex subterraneus subterraneus, Acromyrmex subterraneus molestans*, and *Acromyrmex subterraneus brunneus*, the queens of larger nests showed a smaller number of oviposition than the queens of smaller nests [28]. However, in nests of *Atta sexdens rubropilosa* and *Atta laevigata*, the oviposition of queens from larger nests was superior to that of smaller nests [33,34], similar to the results found in the study. However, we cannot evidence an apparent relation between the oviposition rate and the colony size, due to the methodology adopted in the present research.

The amount of eggs deposited seems to be directly related to the amount of individuals required to maintain the colony [2,3]. However, it is suggested that the queen oviposition rate is probably also related to the symbiotic fungus, required nutritional factors, hormones, and the microclimatic conditions of the nest, which may influence the queens’ behavior, sometimes contributing to a possible stress of these queens in smaller nests.

In this study, the queens were removed from their nests of origin and individualized in plastic containers, where the amount of fungus to feed the colony, the number of workers to perform the grooming of the queen, and the temperature were standardized. Therefore, in this case, due to the fact that the production of eggs is controlled by their endocrine system, what might have influenced the oviposition rate was the food, adequate or not, from the nests of origin.

In relation to the mean of eggs/h in *Acromyrmex rugosus rugosus*, the rate was 2.5, similar to those of the other species of this genus. However, it is important to emphasize that this oviposition behavior of the queens varies greatly within the same species, since Andrade (2002) [28] determined that the average oviposition rate in *A. subterraneus brunneus, A. subterraneus subterraneus*, and *A. subterraneus molestans* was 3.4; 1.6, and 1.2 eggs/h, respectively, during 96 h of oviposition. Marinho and Della Lucia (1998) [2] determined that the oviposition rate of *Acromyrmex crassispinus, A. subterraneus subterraneus*, and *A. subterraneus molestans* queens was 2.5, 2.7, and 3.2 eggs/h, respectively, in this same oviposition period. However, these authors suggested that the posture occurs in pulses, as they found that there are long periods (12 to 24 h) when the queen deprives itself of ovipositing, and then reaches high oviposition rates during a period of up to 12 h. The present study observed a decrease in the number of ovipositions over time. This decrease was also observed by Andrade (2002) [28] in *A. subterraneus* and by Araújo and Della Lucia (1993) [34] in *A. laevigata*, but there is still no biological interpretation to attribute to this fact. 

During the observations, it was found that not all eggs derived from *A. rugosus rugosus* were viable. In *A. subterraneus, A. crassispinus, A. laevigata*, and *A. bisphaerica*, besides viable eggs, non-viable eggs were also observed [2,28,34,35]; the latter are probably used to feed larvae and workers. It is known that, in some species of *Acromyrmex*, workers consume both eggs and larvae [28]. In the present study, it was found that the workers feed on eggs. However, the consumption of larvae and pupae by these insects was not directly observed, but it is probable that this could have occurred, since there was a decrease in the number of larvae and pupae during the research, suggesting the existence of cannibalism in this species.

Thus, in the case of food shortages, the energy expended in the production and care of the offspring can be recovered, serving as an alternative source of energy for the adults, ensuring the success of the colony. Additionally, even though there was a decrease in the number of larvae and pupae, it was deemed necessary for the workers to stay so that they could take care of the offspring. Since the young forms of ants are totally dependent on the care from workers in order to complete the development to the adult stage, the social environment is necessary to induce the larva to eat, to assist in the emergence of the adult, to inhibit the contamination of the offspring, and to care for the fungus garden [17,18].

In addition, in the present study, the eggs, larvae, and pupae were covered by mycelia of the fungus. This mycelial cover placed by the workers on the offspring is notorious in the Attini tribe, and its function is probably to protect immature forms against harmful organisms [8,21], or against predators by promoting the camouflage of the offspring [36]. Therefore, life inside the colony is not always harmonious; however, workers use strategies that favor its balance.

Dijkstra and Boomsma (2006) [12] stated that workers from orphan *Acromyrmex* colonies in the laboratory may fail to produce haploid males if the fungus garden is extremely small, resulting in the starvation of the larvae in the last few instars. There might be a correlation between the availability of food in the colony (symbiotic fungus) and the number of workers to care for the offspring [37].

Food shortages can activate sibling cannibalism in various species of ants. An interesting case is that of the *Linepithema humile* Argentine ant, where workers discriminate between sex, caste, and offspring age, and selectively eliminate larvae according to the social environment [38].

In this study, the average development time of the eggs until the first adult workers of *A. rugosus rugosus* was 62.9 days. Thus, the average development time of these workers is close to that of other species of leaf-cutting ants, ranging from 50 to 69.4 days [6,8,28,30,31,32]. In the three nests studied, there was a great variation in the development time of the larval stage, and consequently in the emergence of the adult, and a small variation in the incubation time of the eggs and of the pupal stage. Although data on life cycle stages are generally from laboratory nests, comparing the data from the present study with the development of initial colonies of *A. sexdens rubropilosa* in the laboratory simulating an environment closer to the natural [6], variation was observed in the incubation, larval, and pupal periods. The duration of each stage in the immature forms was also quite variable when compared to other species of leaf-cutting ants, as shown in Table 2. 

The life cycle duration from egg to adult, besides depending on the ant species, also varies according to environmental conditions, mainly temperature. The higher the temperature, the faster the development [1,8]. In the present work, the immature forms were kept under a controlled temperature, similar to the temperature found in chambers in the natural condition of *Acromyrmex heyeri* (24 °C) [39], in order to avoid fluctuations in the development times of each phase; even so, small variations occurred from one nest to another. According to Bolazzi and Roces (2002) [39], *A. heyeri* workers transport the offspring and the fungus to an environment with preferential temperature, so that their growth is optimized, and this thermal selection that varies from 21 °C to 25 °C is dependent of the initial temperature gradient.

As in the subspecies of the *A. subterraneus* complex [28], in this study not all hatched larvae developed. Although the use of trophic or reproductive eggs in larval feeding has not been observed, it is known that trophic eggs are rich in concentrated protein, which is especially necessary for larval growth, and it is suggested that in their absence, larval development is impaired [40].

In this work, it was not possible to determine larval instars, and other authors have reported this difficulty. Andrade (2002) [28] observed that *A. subterraneus* larvae remained the same size until the sixth day of development, and that after this period they began to change in size day after day, but it was not possible to determine changes in the instars.

Schneider (2003) [20] also did not detect significant alterations in *A. sexdens rubropilosa* larvae, except for the first instar (recently hatched), called microlarva by Hölldobler and Wilson (1990) [1], a phase in which the larva has the size of the egg, and of the latter, when it has already become a pre-pupa. However, Torre-Grossa et al. (1982) [41] verified that the larva of the *Acromyrmex octospinosus* worker goes through four instars, and in the sexual caste the existence of a supplementary instar was also observed. These authors verified that it is possible to determine the instars by dissecting the larvae and observing through microscopy changes that occur in the mesothoracic spiracles and the bristles. Solis et al. (2012) [42] estimated the existence of four larval instars in *Atta sexdens* by a frequency plot of maximum head widths. Worker larvae belonged to two distinct morphological castes: (1) gardeners; and (2) within-nest generalists. However, they used only specimens from founding stage colonies (i.e., lacking adult workers).

*A. rugosus rugosus* workers survived around 3 to 7 months, a period not very different from that of *A. subterraneus*, which had three subspecies studied, for which it was shown that *A. subterraneus subterraneus* workers survived at least 1.5 months and 7 months at most, while those of *A. subterraneus molestans* between 2 and 5.5 months [28], and those of *A. subterraneus brunneus* from 3 to 4 months [22,28]. Nevertheless, the longevity of workers of the *Atta* genus is longer, as there is evidence that they can survive for almost two years. However, the first workers from young nests of *A. sexdens* survived a little less, from 6 to 9 months [8].

Porter and Tschinkel (1985) [43], Chapuisat and Keller (2002) [44], and Camargo et al. (2007) [22] carried out studies on the longevity of workers by size category through the observation of marked ants from their emergence to their death. In *A. subterraneus brunneus* and *Oecophylla smaragdina*, workers’ life expectancy is similar for large and medium-sized ones throughout their lives, whereas the small-sized class has a longer life expectancy compared to the other two classes [22,43]. On the other hand, in *Solenopsis invicta*, the larger the worker the longer its survival [42].

Regarding the workers’ mortality, a high rate was registered in the first weeks in the three nests, meaning a significant reduction in the population. According to Camargo et al. (2007) [22], survival and mortality rates directly influenced the frequencies of behavioral acts (performing a task) in the week when the highest mortality rate and the lowest survival rate were observed, for the three size classes of workers, leading to an abrupt reduction in the frequencies of behavioral acts. This was obviously due to the smaller population contingent of the colonies in question.

## 5. Conclusions

Our study showed that a queen of *A. rugosus rugosus* can deposit more than 100 eggs per day, and that workers can survive for only a few months. On the other hand, we can infer that there is a variation in the oviposition rate of the queen, in the longevity of workers, and in the development time of immature forms, especially in the larval stage.

## Figures and Tables

**Table 1:** Total number of eggs, average oviposition rate (per day) in 96 h and standard deviation (SD), and eggs/h rate of the queens from three laboratory colonies of *Acromyrmex rugosus rugosus.* Temperature: 24 °C. Humidity: 70%.

Nests	Total	Average Rate/Day	SD	Eggs/h
1	338	84.5	53.6	3.5
2	231	57.8	45.7	2.4
3	151	37.8	22.7	1.6

**Table 2:** Average duration of the offspring development stages and average time of worker development (emergence) observed in *Acromyrmex rugosus rugosus* in the present study and in other leaf-cutting ants already studied.

Species	Development Stages/Days			Emergence/Days	References
	Egg	Larval	Pupal	Worker	
*Acromyrmex rugosus rugosus*	17.7	27.4	19.8	62.9	
*Acromyrmex octospinosus*	24.0	18.0	16.0	63.0	[30]
*Acromyrmex lundi*	-	-	-	60.0	[8]
*Acromyrmex subterraneus subterraneus*	21.8	21.8	15.8	62;0	[28]
*Acromyrmex subterraneus brunneus*	21.2	25.2	21.2	69.4	[28]
*Acromyrmex subterraneus molestans*	20.8	17.2	14.2	53.0	[28]
*Atta sexdens rubropilosa*	25.0	22.0	10.0	62.0	[6]
*Atta insularis*	15.5	17.0	14.0	50.0	[31]
*Atta texana*	15.0	-	14.0	50.0	[32]

**Table 3:** Number and maximum and median longevity of workers in three nests of *Acromyrmex rugosus rugosus*. Room temperature of 24 ± 2 °C and relative humidity of 70% ± 20%.

Nest	Number of Workers	Longevity	
		Maximum (Weeks/Months)	Median (Weeks)
1	18	12/3	5
2	27	25/6	5
3	23	29/7	7
